# Development of a Neuropeptide Y-Sensitive Implantable Microelectrode for Continuous Measurements

**DOI:** 10.1021/acssensors.4c00449

**Published:** 2024-05-06

**Authors:** Lauren Fernández-Vega, Dorian Enid Meléndez-Rodríguez, Mónica Ospina-Alejandro, Karina Casanova, Yolimar Vázquez, Lisandro Cunci

**Affiliations:** † Department of Chemistry, University of Puerto Rico-Rio Piedras, 17 Ave Universidad Ste 1701, San Juan, Puerto Rico 00931, United States; ‡ Department of Chemistry, Universidad Ana G. Méndez, Carr. 189, Km 3.3, Gurabo, Puerto Rico 00778, United States

**Keywords:** neuropeptide Y, aptamer biosensor, intermittent pulse amperometry, square wave voltammetry, dynamic measurement

## Abstract

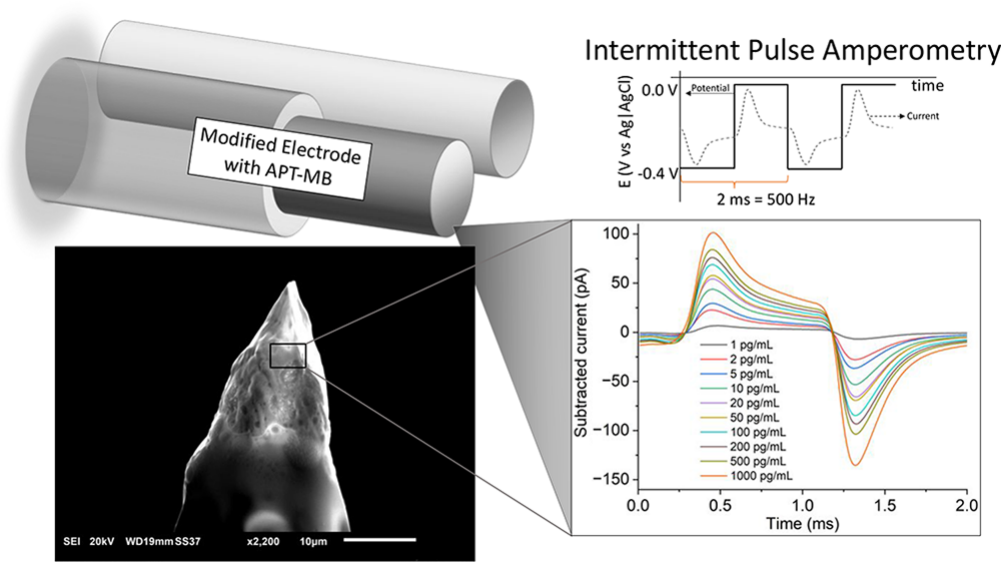

In this work, we present the development of the first implantable aptamer-based platinum microelectrode for continuous measurement of a nonelectroactive molecule, neuropeptide Y (NPY). The aptamer immobilization was performed via conjugation chemistry and characterized using cyclic voltammetry before and after the surface modification. The redox label, methylene blue (MB), was attached at the end of the aptamer sequence and characterized using square wave voltammetry (SWV). NPY standard solutions in a three-electrode cell were used to test three aptamers in steady-state measurement using SWV for optimization. The aptamer with the best performance in the steady-state measurements was chosen, and continuous measurements were performed in a flow cell system using intermittent pulse amperometry. Dynamic measurements were compared against confounding and similar peptides such as pancreatic polypeptide and peptide YY, as well as somatostatin to determine the selectivity in the same modified microelectrode. Our Pt-microelectrode aptamer-based NPY biosensor provides signals 10 times higher for NPY compared to the confounding molecules. This proof-of-concept shows the first potential implantable microelectrode that is selectively sensitive to NPY concentration changes.

Nonelectroactive molecules in the brain are currently unable to be measured with high temporal and spatial resolution. The understanding of brain chemistry in space and time has been greatly advanced by the development of fast-scan cyclic voltammetry and amperometry, which allowed researchers to study subsecond processes related to electroactive molecules such as catecholamines. In the past decade, enzymatic electrodes have been developed using these two techniques to measure nonelectroactive molecules for which oxidases exist, taking advantage of their catalytic reaction.^1−11^ However, there is still a universe of nonelectroactive molecules for which no methods are available: Neuropeptides. This is due to the fact that the chemical environment where they are released has different confounding molecules that may interfere with their measurements. Due to their structural differences compared to classic neurotransmitters and the structural similarity between neuropeptides, the methods currently used for other molecules do not work for their detection. Thus, there is an evident lack of technology with the spatial and temporal resolution needed for in situ measurements of neuropeptides and conventional neurotransmitters at the same time in order to understand their implications in neurological diseases such as anxiety.

Detection of nonelectroactive molecules has been the focus of research for many years, showing the importance of this area of work. In 2008, Lu et al.^12^ developed an aptamer-based electrochemical sensor for thrombin without the need for conformational changes using ferrocene as the redox label. Furthermore, Jiang et al.^13^ from the same research group (Dr. Lanqun Mao’s group) developed an aptamer superstructure-based electrochemical sensor for the detection of adenosine triphosphate (ATP) as part of a microdialysis system. ATP is a nonelectroactive molecule that could not be measured using typical microdialysis systems with electrochemical detectors, therefore advancing the difficult measurement of nonelectroactive molecules. Using SWV, Mao’s group was able to measure ATP electrochemically with time resolution in the order of minutes. Santos-Cancel et al.^14^ also worked on the measurement of ATP and tobramycin using aptamers taking advantage of IPA. IPA increased the time resolution in the measurement to more than a thousand times per second. It is noteworthy that these works were all done using planar electrodes that cannot be implanted into tissue due to their size. In this work, we are giving one more step in the advancement of aptasensors by demonstrating the first implantable microelectrode that can measure nonelectroactive molecules with high temporal and spatial resolution. Aptamers have been previously used in carbon fiber implantable microelectrodes for the detection of electroactive molecules such as dopamine. In 2020, Hou et al.^15^ demonstrated a novel and generalizable method for measuring neurotransmitters in vivo using aptamers with carbon fiber microelectrodes increasing the stability. Li et al.^16^ demonstrated lower biofouling, even higher stability, and increased selectivity for dopamine measurements provided by the aptamer modification. This was achieved by developing a novel method for carbon fiber modification with higher aptamer density on their surface, overcoming a known challenge also shown previously by our group.^17^ In the past few years, Mao’s group has shown the stability of aptamer-modified microelectrodes in tissues. Therefore, our work is focused on combining the selectivity and stability of aptamers shown in carbon fiber microelectrodes for electroactive molecules and in planar electrodes for nonelectroactive molecules by using aptamer-modified implantable platinum microelectrodes for the measurement of NPY.

NPY, the most abundant neuropeptide in the brain,^18,19^ regulates a wide range of biological processes, including mood regulation, feeding behavior, learning and memory, circadian rhythms, and pain perception.^18,20^ NPY has been implicated in multiple human diseases, including obesity, alcoholism, and depression.^21−23^ NPY has robust anxiolytic and antidepressive properties,^21,24,25^ and reduced NPY is involved in anxiety disorders, post-traumatic stress disorder, and depression.^21,26,27^ Despite its importance, there is currently no method for measuring NPY in the brain with high temporal and spatial resolution. It is essential to fill this technological gap to fully understand the mechanisms by which NPY regulates brain circuits, enabling the development of pharmacological tools to attack NPY-related diseases. Until this understanding is brought to neurobiological researchers, the connections between behavior, neurotransmitter release, and NPY release will continue to lag.

Recently, Seibold et al. reported the first use of aptamer-modified microelectrodes to measure NPY dynamically, which has the potential to have excellent spatial and temporal resolution.^28^ The authors were able to measure NPY in serum very accurately. However, measurements were only possible as low as 20 nM (4704 pg/mL), which is much higher than needed for brain measurements. Churcher et al. reported a detection limit of NPY of 10^29^ and 50 pg/mL^30^ in sweat using antibodies and larger electrodes. This is a significant advance in the field, with the main issue being the intrinsic disadvantages of antibodies that are not stable for long-term use and the fact that these electrodes cannot be used in the brain due to their size.

Herein, we present the development of the first implantable aptamer-based platinum (Pt) microelectrode to detect NPY selectively, a nonelectroactive molecule. Platinum microelectrodes were chosen based on previous results on antibiofouling properties in the presence of aptamers^17^ and the current investigations performed in deep brain stimulation using commercially available platinum microelectrodes.^31−33^ Using SWV as our proof-of-concept in our steady-state measurements, we compared three NPY aptamers. After choosing the sensor with the best performance, we performed dynamic measurements using the IPA technique.^14^

To optimize the aptamer for measuring MB-ending aptamers in implantable microelectrodes, we used SWV to test three aptamers: 4.31, 4.20, and 4.31, which have dissociation constants spanning from 0.2 to 1.0 μM.^17,34^ We showed the adsorption of NPY on aptamer-modified implantable microelectrodes previously using electrochemical impedance spectroscopy (EIS).^17^ Although EIS is a great technique to measure changes in the surface of microelectrodes, it is slow for dynamic measurements due to the measurement of the entire spectrum. The form factor of the implantable microelectrodes changes the diffusion from one dimension in planar electrodes to semispherical in our implantable microelectrodes. The electrochemistry is expected to change due to an increased diffusion of NPY toward the electrode for faster equilibrium.

We were able to detect NPY selectively over three potential confounding peptides, pancreatic polypeptide (PP), peptide YY (PYY), and somatostatin (SOM), at a rate of 500 Hz. Our results showed the feasibility of using this strategy for localized measurements in the brain.

## Experimental Section

### Fabrication of Platinum Microelectrodes

Platinum fiber microelectrodes used in this work had a diameter of ≈20 μm and were fabricated using a previously published procedure.^17^ Briefly, the microelectrodes were sealed using a borosilicate glass capillary (1.5 mm × 0.86 mm). A single platinum wire was aspirated into the glass capillary using a vacuum. Then, microelectrodes were placed in the micropipette puller (Narishige PC-10) to obtain the electrode. Afterward, the platinum microelectrode with the tip exposed approximately 15–60 μm was put back into the micropipette puller to seal the platinum tip in the borosilicate capillary. The electrical connection was made with silver paint and a silver-plated copper wire connected to the potentiostat for measurements. Finally, platinum microelectrodes were cleaned electrochemically in 0.5 M H_2_SO_4_ vs Ag|AgCl at 100 mV/s for 240 cycles.

### Reagents

Electrochemical characterization was carried out in artificial cerebrospinal fluid (aCSF) prepared at pH 7.4, as reported previously.^20^ Neuropeptide Y (GenScript) was diluted in aCSF to prepare standard solutions. The final solutions were achieved by adding the standard solutions to the initial 15 mL of aCSF. The detection of NPY was done using different concentrations by adding NPY at the end of each run to prepare target concentrations between 1 and 1000 pg/mL^34^ to understand the behavior of the microelectrodes at different concentrations. The electrochemical cell was stirred for 1 min without disturbing the electrodes after adding each aliquot of NPY and waiting until the solution was stagnant after stirring to avoid mass transfer due to convection. For dynamic measurements, solutions were prepared fresh daily. A stock solution was prepared at 1,000 pg/mL in aCSF, and concentrations from 500 to 1 pg/mL were prepared by serial dilutions using the same buffer.

### Electrochemical Measurements

All electrochemical measurements were done using two (dynamic measurements) or three-electrode (static measurements) electrochemical cells. In the three-electrode configuration, a platinum fiber microelectrode served as the working electrode, a platinum wire as the counter electrode, and an Ag|AgCl reference electrode (3 M NaCl-filled solution). A platinum fiber microelectrode served as the working electrode for the two-electrode configuration, and a silver wire treated in chloride solution as the pseudoreference electrode. Static measurements were done using a three-electrode electrochemical cell with a capacity of up to 15 mL (Prod. MF-1084, Bioanalytical Systems, Inc.). Reference 600+ Gamry potentiostat was used for electrochemical measurements, and all data were collected using Gamry Instruments Framework software. All potentials in this work are expressed versus Ag|AgCl. From −0.4 to 0.0 V, a frequency of 30 Hz was used for SWV, and for cyclic voltammetry, a current range from −0.2 to 0.6 V was used at a scan rate of 50 mV/s. All experiments were done inside a Faraday cage. Gamry Echem Analyst, OriginPro, and Matlab software were used to analyze all the data.

Dynamic measurements were carried out in a custom-made two-electrode setup flow cell. A 2-in. rod of Teflon was machined to have a 1.5 in. diameter ×1 in. depth reservoir in which a 1/16 in. PEEK tubing entered from below. On the side, a hole was made so the solution would overflow to a waste recipient placed below the cell. The tip of the microelectrodes was positioned as far inside the PEEK tubing as possible using a micromanipulator. The inlet of the flow cell was connected to a six-port valve (VICI Cheminert) with a 100-μL loop where NPY solutions were introduced before being pushed to the cell by the buffer when the valve was actioned. An HPLC pump at 1 mL/min introduced the buffer to the flow cell. WaveNeuro Potentiostat from PINE Research with a single channel was used to perform the measurements. A stabilization time of the electrode signal was carried out before each measurement.

### Physical Characterization

Scanning electron microscopy (SEM) (JEOL JSM-6010LA) was used for electron microscopy to observe and measure the microelectrode surface.

### Aptamer Immobilization with Methylene Blue as a Redox Label

**Figure 1:**
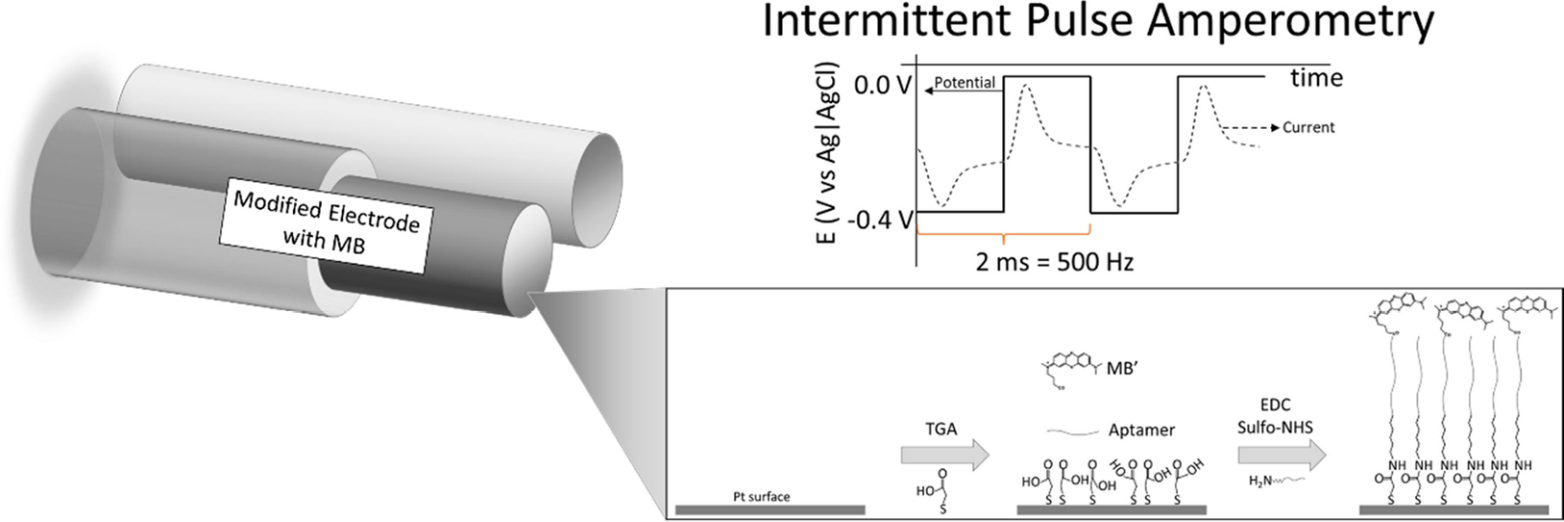
Scheme of surface modifications in Pt microelectrodes.

Figure 1 shows the modification of the microelectrodes. The Pt microelectrodes were modified due to the possibility of forming a Pt–S strong bond using thiol-terminated aptamers. Microelectrodes were modified by dipping into 1.0 μM solutions of single-stranded DNA aptamers for 24 h at room temperature. The following aptamers were tested:

Aptamer 4.31: (5′-O–(CH_2_)_6_–S–S–(CH_2_)_6_–O–PO_3_– AGC AGC ACA GAG GTC AGA TGC AAA CCA CAG CCT GAG TGG TTA GCG TAT GTC ATT TAC GGA CCT ATG CGT GCT ACC GTG AA −O–CH_2_–CHOH–CH_2_–O–(CH_2_)_3_–NH_3_-3′); (*K*_D_= 0.3 ± 0.2 μM).^34,35^

Aptamer 4.20: (5′-O–(CH_2_)_6_–S–S–(CH_2_)_6_–O–PO_3_–AGC AGC ACA GAG GTC AGA TGC GTT TTT TAC GGC TGG CAA CGG AAG TCA GTT TAT TCT TCA CCT ATG CGT GCT ACC GTG AA–O–CH_2_–CHOH–CH_2_–O–(CH_2_)_3_–NH_3_-3′); (*K*_D_= 0.8 ± 0.3 μM).^34,35^

Aptamer 4.13:5′-O–(CH_2_)_6_–S–S–(CH_2_)_6_–O–PO_3_–AGC AGC ACA GAG GTC AGA TGT GTT AGG CTG GGA CAC GTA TTA CAC TTA CCG TAA ATA TTT CCT ATG CGT GCT ACC GTG AA–O–CH_2_–CHOH–CH_2_–O–(CH_2_)_3_–NH_3_-3′); (*K*_D_= 1.0 ± 0.3 μM).^34,35^

Functionalized aptamers were bought from Integrated DNA Technologies (IDT Technologies). After being attached to the Pt surface, the exposed carboxylic acid at the 3′-end was used to attach a MB molecule as redox-label by dipping in a 1.0 μM solution MB-NHS ester (ATTO-TEC) to allow the formation of an amide bond.

### Statistics

All of the data was analyzed by Gamry Echem Analyst, OriginPro, and Matlab software. OriginPro 2022b was used for statistical analysis. All data are provided as mean ± SD, and the significance is defined as *p* ≤ 0.05.

## Results and Discussion

### Pt Microelectrode Characterization

We tested our modified microelectrodes with different concentrations of NPY in steady-state measurements to ensure that we could measure them using SWV and find the limit potentials for IPA. We tested three aptamers based on their affinity to NPY and previously published results that showed high affinity for NPY and against PYY, which has a similar structure. All characterization steps were performed with each aptamer modification.

In order to modify the Pt microelectrode, we reduced the 5′-end to have a thiol-free group available for binding. Using affinity-based chemistry, Pt–S bonds are formed, allowing the aptamer to stay attached to the microelectrode surface (Figure 1). We have shown a good affinity between aptamer-modified Pt microelectrodes with NPY in previous work using this modification procedure.^17^ In this case, the 3′-end underwent an amidation reaction to conjugate a methylene blue (MB) derivative (NHS-ester-terminated) as our redox label, as shown in Figure 1. Seibold et al. also reported different aptamer–MB constructs in which the aptamer is internally and terminally MB-labeled. Their results showed that the terminally MB-label aptamer had a better performance in measuring the presence of NPY.^28^

**Figure 2:**
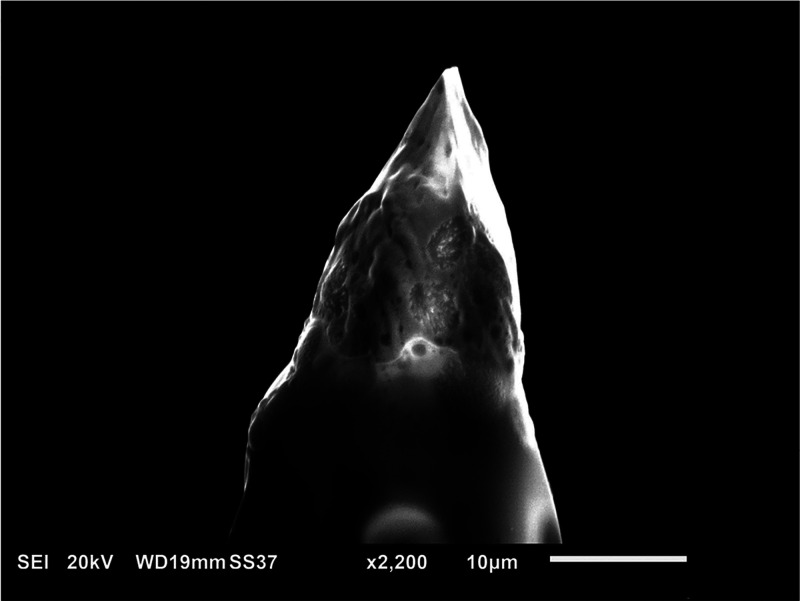
SEM on fabricated Pt microelectrodes.

Physical characterization was performed using SEM (Figure 2) to observe the surface of the microelectrodes. Figure 2 shows a nonmodified microelectrode where we can observe the size of the exposed platinum tip at 15 by 20 μm. The rough structure of the platinum is due to the fabrication procedure in which the platinum wire of 25 μm diameter is elongated until rupture. Therefore, due to the high ductility of the platinum, the final tip is smaller than 25 μm in size, showing a typical ductile fracture. The heat treatment of the borosilicate glass after microelectrode breaking provides a good seal around the metal, as seen in Figure 2. SEM was also used to observe aptamer-modified microelectrodes but no discernible difference was found; therefore, it is not shown in this paper.

**Figure 3:**
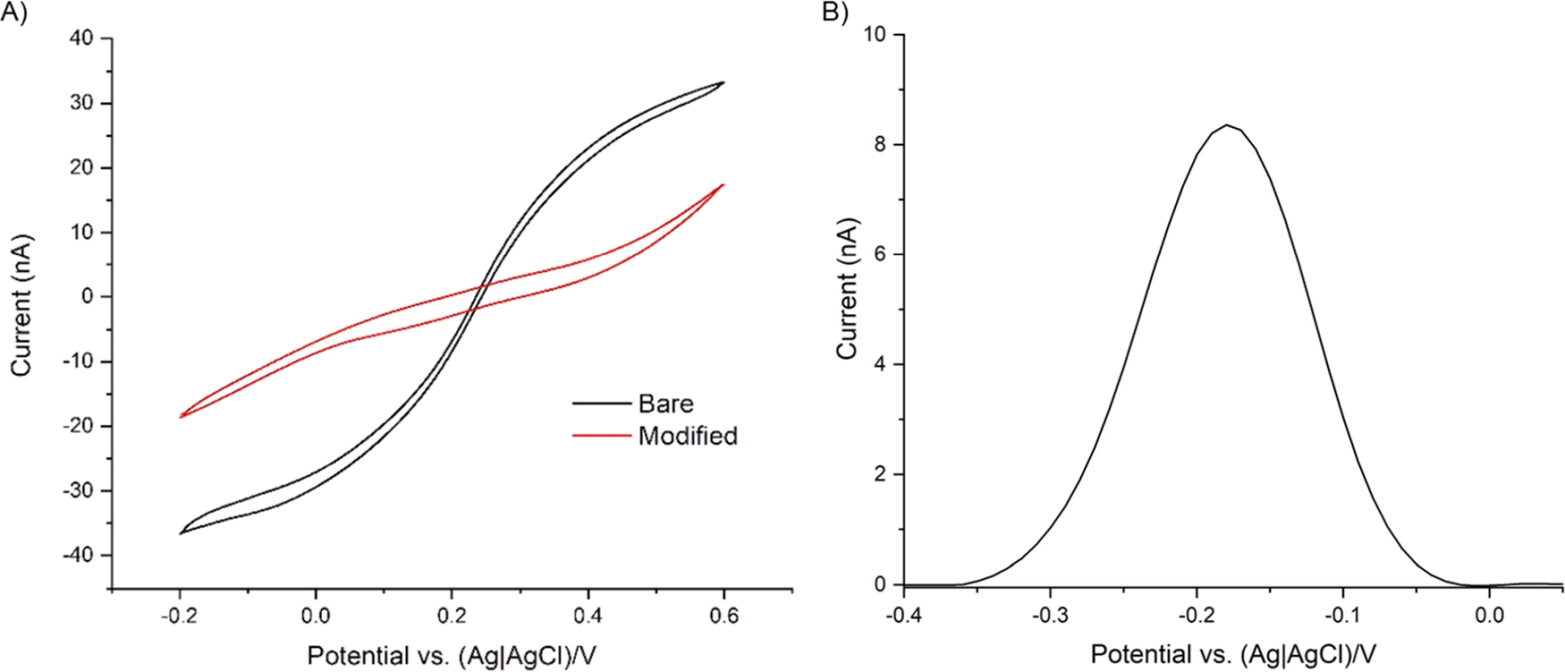
Electrochemical characterization of microelectrode surface modifications (A) before (black) and after (red) aptamer modification in 5 mM K_3_[Fe(CN)_6_]/K_4_[Fe(CN)_6_] in KCl at 50 mV/s scan rate and (B) after methylene blue modification in aCSF (pH = 7.4) from −0.4 to 0.0 V at a frequency of 30 Hz using SWV.

Electrochemical characterization was conducted in a three-electrode cell with a solution of 5 mM K_3_[Fe(CN)_6_]/K_4_[Fe(CN)_6_] in 0.1 M KCl. Using cyclic voltammetry, we confirmed the typical behavior of the microelectrode as seen in Figure 3A, before (black line) and after (red line) aptamer immobilization. Through this experiment, we could establish if the modification occurred due to the change in the electron transfer between the modified microelectrode and the [Fe(CN)_6_]^3–^/ [Fe(CN)_6_]^4–^ redox pair in the solution. Due to the surface blockage generated by the aptamer, we observed an increased electron transfer resistance (Figure 3A, red line) compared to the bare electrode (Figure 3A, black line) between the solution and the electrode. For the final step of the microelectrode modification, MB conjugation at the 5′-end, the characterization was performed in aCSF at pH 7.4 using SWV from −0.4 to 0.0 V at a frequency of 30 Hz. As expected, the MB signal showed a peak close to −0.2 V versus Ag|AgCl, as shown in Figure 3B.

### NPY Measurements Using SWV in Standard Solutions

After fabrication and characterization procedures, steady-state measurements were performed to test the feasibility of our sensor to detect NPY in an artificially relevant buffer such as aCSF. Ten concentrations were tested from 1 to 1000 pg/mL for the 4.31, 4.13, and 4.20 aptamers against NPY, PP, PYY, and SOM. NPY, PYY, and PP are members of the NPY family of peptides with 36 amino acids each. Of the 36 amino acids, PYY shares 25 of the amino acids in the same position as NPY, and PP shares 17. This makes it very important to measure the selectivity of NPY measurements against PYY and PP. We also tested SOM because of its presence in the same brain regions where NPY is found since NPY can be released in subsets of SOM-expressing interneurons.^36−38^

**Figure 4:**
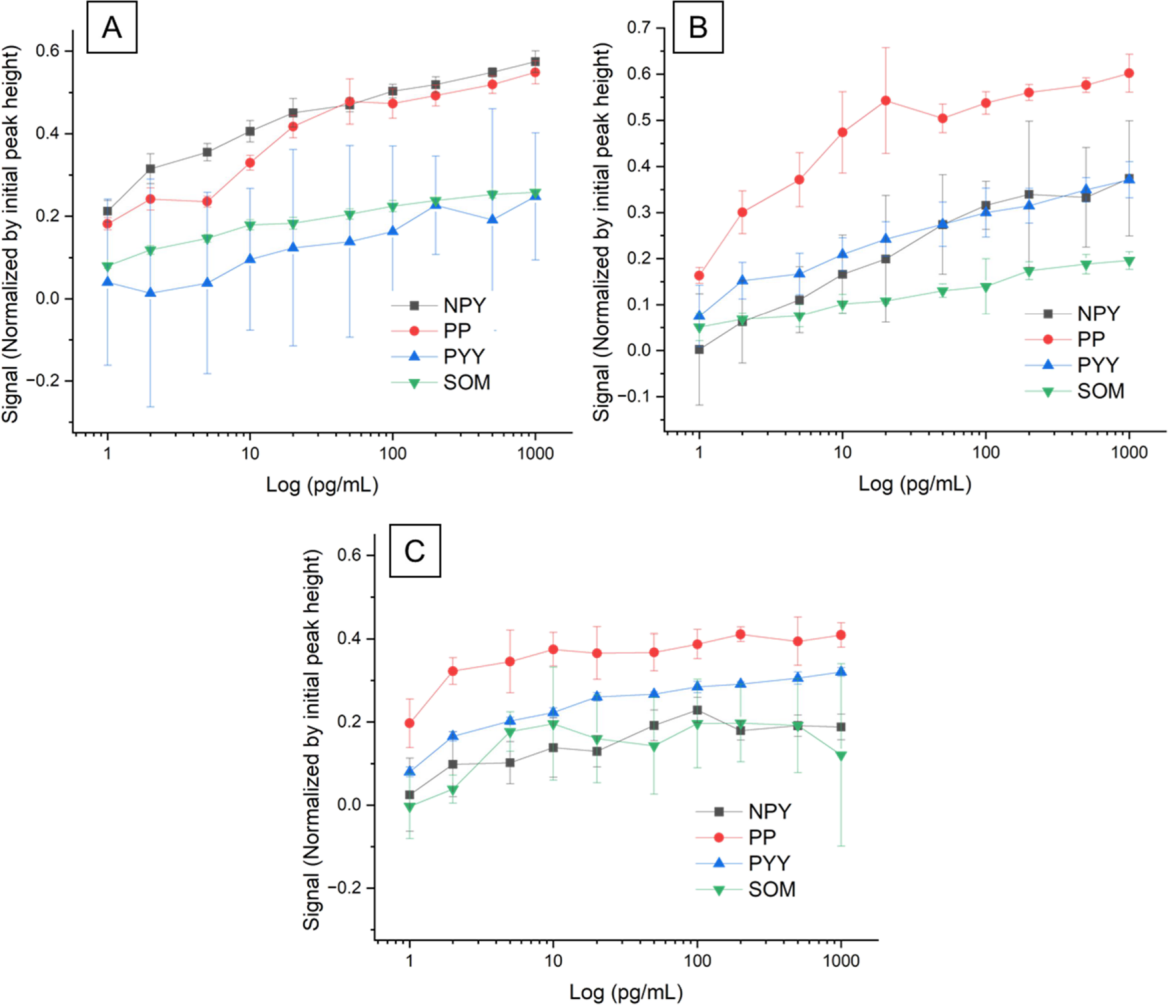
NPY measurements using SWV in standard solutions. (A) Aptamer 4.31, (B) Aptamer 4.13, and (C) Aptamer 4.20.

Microelectrodes are fabricated by hand and have different tip lengths and defects depending on how the pulling phase affects the Pt wire. Even environmental factors such as humidity affect the way and conditions in which implantable microelectrodes are made. As with carbon fiber microelectrodes, each of these implantable platinum microelectrodes is different. Using different microelectrodes, we performed all SWV experiments normalizing by the height of the initial peak at zero concentration and measuring the change in height for each electrode. After standardization, we could have all the results on the same scale to compare their performance against NPY and the other three peptides (Figure 4).

We could observe how different the three aptamers behave in our sensor against NPY. The dissociation constants of aptamers 4.31, 4.13, and 4.20 are 0.3 ± 0.2 μM, 0.8 ± 0.3 μM, and 1.0 ± 0.3 μM, respectively. If we only focus on the black line (Figure 4) that refers to adding standard solutions of NPY, results showed that, as the aptamer dissociation constant increases, the NPY response decreases, with the aptamer 4.31 as the best NPY response of all three sensors. In terms of selectivity, comparing their response against PP, PYY, and SOM, only aptamer 4.31 showed a higher response toward NPY than the other peptides. Moreover, in the aptamer 4.31, there is no significant difference between NPY and PP. These could be due to their similarity in structure and possibly the aptamer binding in the same region, which did not occur with PYY. However, it is hypothesized that fast dynamic measurements could provide selectivity between NPY and PP due to their different adsorption rates in these aptamers and their different *K*_D_ causes. In physiological conditions, the exposition time will be short since the liberation of neuropeptides occurs within the subseconds range. Additionally, biological fluids are in constant movement in which case dynamics measurements are expected to represent biological measurements. In addition, the use of different electrodes can provide a confounding variable because, even with normalization, they are going to have higher errors due to structural differences between electrodes. As it is known with carbon fiber microelectrodes for neurotransmitter measurements, the microelectrode used for the experiments must be calibrated after using a flow cell to ensure correct concentration measurements.^39−42^

Based on the results obtained with SWV and the results published previously by us^17^ and other research reports,^28^ the aptamer 4.31 was the best for dynamic measurements.

### Optimization of Intermittent Pulse Amperometry Frequency

SWV is a pulse voltammetry technique that allows quick measurements with good sensitivity compared to other pulse and sweep techniques. However, it lacks enough speed for “in-brain” measurements. In order to increase the speed of SWV measurements, frequency has to be increased (step duration has to be decreased); therefore, the faster the technique is applied, the less faradaic current is seen, and the nonfaradaic currents dominate the data. This is an impediment for the SWV technique to go faster than a few scans per second. IPA, on the other hand, applies a square wave with the limit potentials on both sides of the SWV peak.^14,28^ By doing this, the current measured during each half of the square wave applied measures the oxidation and reduction currents, respectively. An electrical current peak that corresponds to nonfaradaic processes (ionic currents) is expected to be observed, with a decrease that continues into faradaic currents. The area under these curves provides the total charge of the faradaic and nonfaradaic processes in the electrode during the IPA. Figure 1 shows the overlap of the potential applied and the current measured using IPA. IPA provides a faster technique to measure changes in the surface of the electrode, with a high potential to be used for dynamic measurements in implantable microelectrodes.

**Figure 5:**
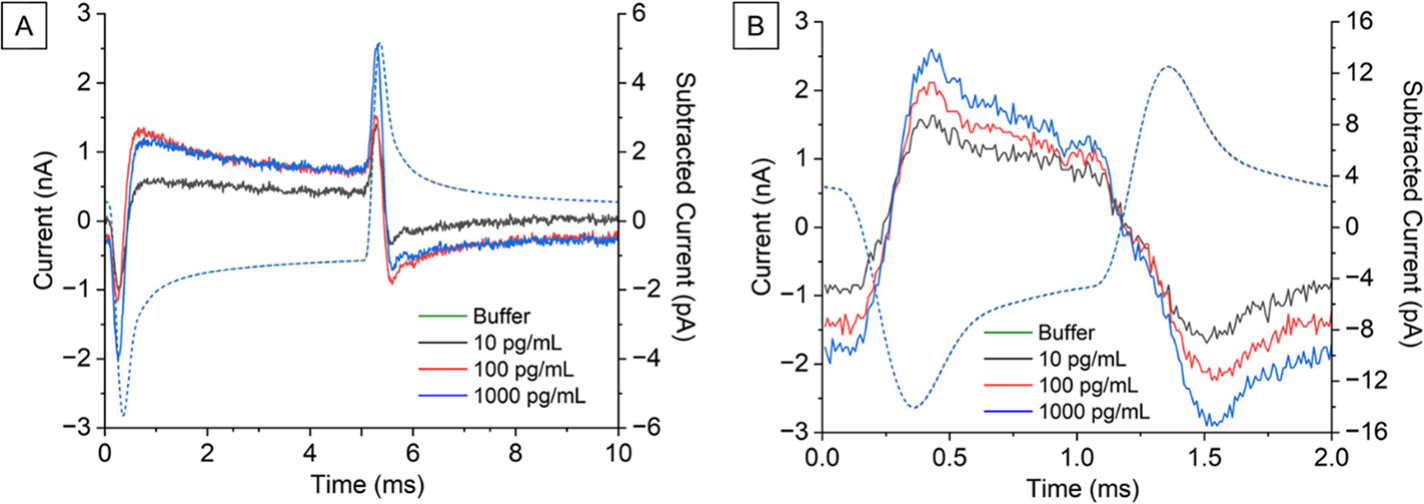
Intermittent pulse amperometry waveform and analysis at the different NPY concentrations at (A) 100 and (B) 500 Hz. Dashed line is the data for the buffer, 10, 100, and 1000 pg/mL NPY without subtraction. Solid lines are the data after buffer subtraction at 10, 100, and 1000 pg/mL NPY.

Dynamic measurements were performed using IPA in a two-electrode setup. As stated before, speed becomes critical when our goal is to measure in the brain with high spatial and temporal resolution. Until now, this has not been possible with any current technique; therefore, we decided to use IPA from −0.4 to 0.0 V following the potentials measured in SWV (Figure 3B). Data were measured by applying a square wave using WaveNeuro with HDCV software. Matlab was used to convert the data obtained in.hdcv files that are saved in a 32-bit, floating-point format IEEE 754 standard and process it. After trying several perspectives of processing our data, we could see the changes over the course of the experiment. We measured frequencies of 100 and 500 Hz to test the extent to which it was necessary to wait more time in the amperometry to decrease the nonfaradaic current and have the faradaic current more dominant in the measurements. As can be seen in Figure 5, the data obtained at both frequencies when injecting the buffer gave us a response very similar to the injected concentrations (1, 10, and 1000 pg/mL, dashed lines). The four curves cannot be separated between them by eye since they are all overlapping. The data can be separated in the reduction (first half) and oxidation (second half) of MB. Within each part, the peak is due to the sum of the faradaic and nonfaradaic currents. The nonfaradaic current is anticipated to exhibit a more rapid decline compared to the faradaic currents within semispherical microelectrodes. In scenarios involving the redox-label present in solution at a constant concentration, the faradaic currents are expected to decrease to a stabilized nonzero value. However, in this case where the number of MB molecules on the microelectrode surface is limited, the faradaic currents should approach zero due to this constraint. Therefore, the peak is expected to be mainly composed of nonfaradaic processes (ionic current), while the decrease in current to a semisteady state should be composed mainly of faradaic currents. Interestingly, when we subtracted the signal corresponding to the buffer in all the concentrations, even small changes at 10 pg/mL were distinguishable from the higher concentrations. Figure 5A shows the measurement of 10, 100, and 1000 pg/mL (solid lines) after buffer subtraction obtained using IPA at 100 Hz. There was no difference in signal between 100 and 1000 pg/mL, probably due to saturation of the microelectrode with NPY. When using this data as the analytical signal for our sensor, there is a compromise between using the first data points that correspond mainly to nonfaradaic processes or using the latter data points where the faradaic process of MB oxidation/reduction is happening, but lower currents are measured losing sensitivity.

Differently, at 500 Hz, we could see a differentiation between the same three concentrations, a clear definition of the nonfaradaic peak, and a decrease toward lower faradaic currents. Figure 5B shows the data obtained at 10, 100, and 1000 pg/mL using IPA at 500 Hz after buffer subtraction (solid lines). Comparing the data obtained at these two frequencies, we defined 500 Hz to be the optimum frequency for our implantable Pt microelectrodes in this concentration range. The maximum separation between the concentrations (Figure S1) was found at the oxidation and reduction peaks; therefore, our analytical signal was defined as the subtraction between the maximum and the minimum to compensate for any error due to the drifting of the signal.

Additionally, there is a peak at 100 Hz at the beginning of each potential pulse that does not appear at 500 Hz. During each half of the square wave applied, there is a nonfaradaic current that corresponds to the ionic movements and a faradaic current that corresponds to the electron transfer. The nonfaradaic current tends to be of shorter duration than the faradaic current. During one-half of the square wave applied (e.g., −0.4 V), specific ions move toward the working electrode, generating a nonfaradaic current. At the application of the other half of the square wave (e.g., 0.0 V), there is an opposite movement of the ions generating a contrary nonfaradaic current. When using 100 and 500 Hz, the duration of each amperometry section lasts 10 and 2 ms, respectively. Consequently, at 100 Hz, a duration five times longer should move and accumulate more ions closer to the working electrode that will generate a larger ionic current at the potential switching. Furthermore, because this is a signal-off sensor, the faradaic current corresponding to the MB redox reaction opposes the potential application with increased NPY concentration. Therefore, we hypothesize that at 100 Hz, the measured ionic current is higher than the decrease in electron transfer due to the presence of NPY, explaining the additional peak observed that opposes the electron transfer due to the MB redox reaction.

### Dynamic Measurements of NPY Using IPA

**Figure 6:**
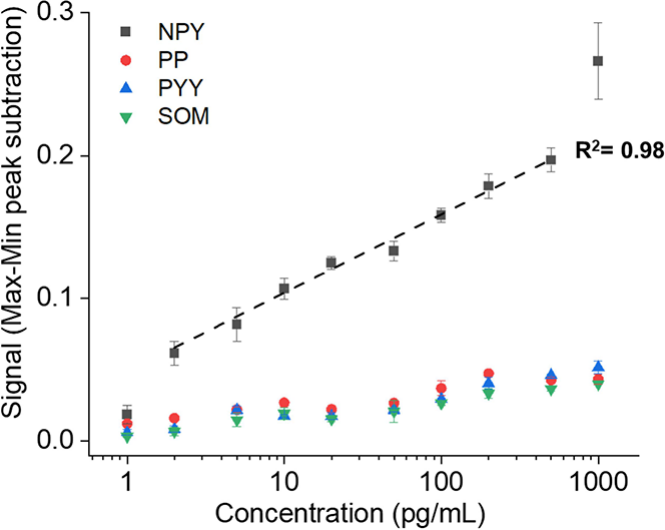
Calibration curve for NPY, PP, PYY, and SOM after buffer subtraction. Every measure was performed in a *n* = 4 for NPY and *n* = 3 for PP, PYY, and SOM, and the final value was obtained by subtracting the maximum and the minimum value in every waveform plot.

Finally, dynamic measurements (1–1000 pg/mL) were performed in an *n* = 4 for NPY and *n* = 3 for PP, PYY, and SOM on the same modified microelectrode at 500 Hz (Figure 6). Raw data for the continuous measurements are shown in the Supporting Information (Figure S2). The same data analysis was performed as shown in Figure 5. The data were processed, the maximum and minimum values for each waveform for each concentration were subtracted, and the standard deviation was calculated from the repetitions in each concentration to obtain Figure 6. We measured concentrations as low as 1 pg/mL with high selectivity starting at 2 pg/mL. NPY data was about 10 times higher compared to the other peptides, as observed in Figure 6 (black line), compared to PP (red), PYY (blue), and SOM (green) lines. Dashed line shows the linear regression between 2 and 500 pg/mL where it follows a linear relationship with *R*^2^ = 0.98. The stability of the microelectrodes was studied by running one last NPY concentration of 1000 pg/mL through the flow cell after the measurement of SOM. This last measurement of 1000 pg/mL was within the error limits of the data obtained for 1000 pg/mL.

## Conclusions

Physical and electrochemical characterization showed a successful surface modification on the Pt microelectrodes using EDC/Sulfo-NHS ester coupling chemistry in a rough semispherical surface. By steady-state measurements and compared with previous publications by our research group and other researchers,^17,28^ it was possible to determine the best aptamer for the dynamic measurements of NPY, which was the 4.31 aptamer developed by Mendonsa et al.^34^ with a *K*_D_ = 0.3 ± 0.2 μM, the lowest of the aptamers tested. The optimization of the IPA frequency was carried out by comparison of the differences in each of the NPY standard solution concentrations. At 500 Hz, all concentrations were clearly differentiated compared to the measurements performed at 100 Hz. Dynamic measurements were performed using the 4.31 aptamer at a 500-Hz frequency against NPY, PYY, PP, and SOM continuously in the same microelectrode. Data results showed a clear selectivity, about 10 times higher signal toward NPY than the other three neuropeptides. Our results demonstrate for the first time the development of a potentially implantable and selective NPY biosensor with a high temporal and spatial resolution with a tip of ca. 15 by 20 μm.

## Supporting Information Available

Buffer-subtracted and raw data obtained measuring NPY and other similar molecules (PDF)
