# Supplementary material for: Development of a Neuropeptide Y-Sensitive Implantable Microelectrode for Continuous Measurements
Source: ACS Sens. 2024 May 6;9(5):2645–52. doi: 10.1021/acssensors.4c00449 (PMC11127761; doi:10.1021/acssensors.4c00449)
Supplement: Supplementary file 1 — se4c00449_si_001.pdf [file se4c00449_si_001.pdf]

Supporting Information

Development of an NPY-sensitive Implantable Microelectrode for Continuous Measurements

Lauren Fernández-Vega [b], Dorian Enid Meléndez-Rodríguez [b], Mónica Ospina-Alejandro [b], Karina Casanova[b], Yolimar Vázquez [b], Lisandro Cunci[a,*]

[a] Department of Chemistry, University of Puerto Rico – Rio Piedras, 17 Ave Universidad Ste 1701, San Juan, Puerto Rico 00931, United States
[b] Department of Chemistry, Universidad Ana G. Méndez, Carr. 189, Km 3.3, Gurabo, Puerto Rico 00778, United States

Corresponding Author
* Tel.: +1-787-764-0000 x1-88554; E-mail address: lisandro.cunci@upr.edu (L. Cunci).

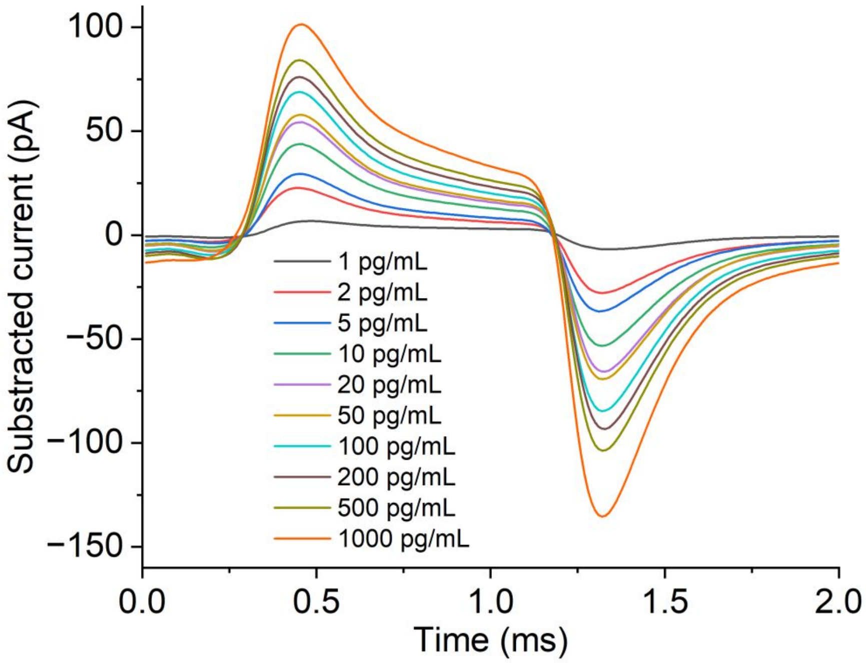

Figure S1 – Buffer subtracted data obtained measuring NPY at concentrations between 1 and 1,000 pg/mL in a flow cell showing the differences between concentrations in the analytical signal.

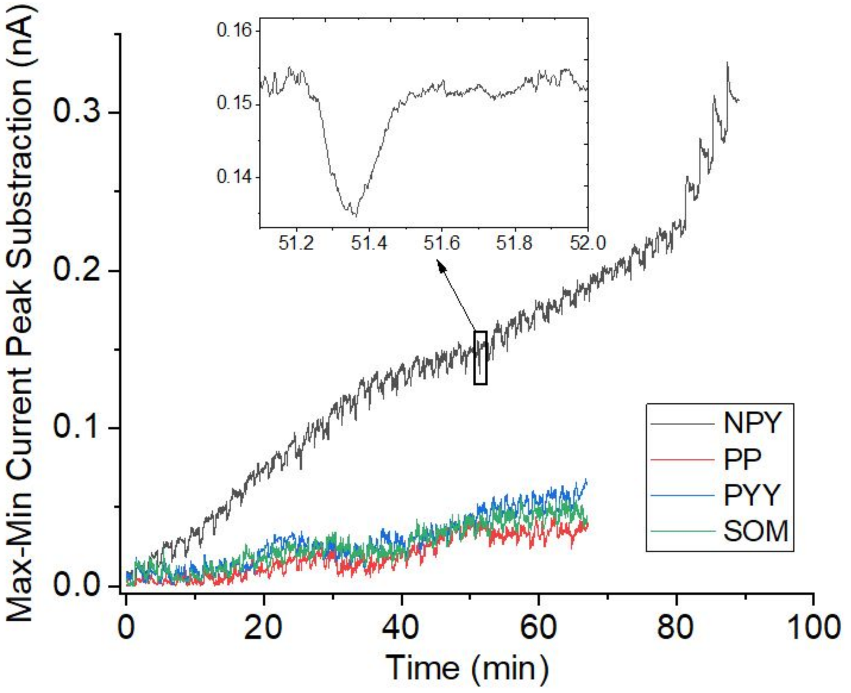

Figure S2 – Buffer subtracted continuous measurements of NPY, PP, PYY, and SOM using the same microelectrode at concentrations between 1 and 1,000 pg/mL in a flow cell.
